# Can Trapping Abundance Data Be Used to Identify Persistent Target Areas for *Culicoides* Biting Midge Control Efforts?

**DOI:** 10.3390/insects17060653

**Published:** 2026-06-20

**Authors:** Aaron M. Lloyd, Daniel L. Kline, Karen E. McKenzie, Daniel A. Hahn

**Affiliations:** 1 Lee County Mosquito Control District, Lehigh Acres, FL 33971, USA; 2 United States Department of Agriculture-Agricultural Research Service-Center for Medical, Agricultural and Veterinary Entomology, Gainesville, FL 32608, USA; 3 Contract Entomologist, Melbourne, FL 32904, USA; 4 Entomology and Nematology Department, University of Florida, Gainesville, FL 32611, USA; dahahn@ufl.edu

**Keywords:** *Culicoides furens*, biting midges, Mosquito Magnet^®^ Liberty Plus, residential island, Florida

## Abstract

Florida mosquito control districts are increasingly confronted with severe *Culicoides* biting midge problems in coastal areas. Yet, there is no clear guidance for integrating *Culicoides* management into mosquito-focused operations. This study describes population abundance and distribution trends for the biting midge *Culicoides furens* on a residential island in Cedar Key, Florida. We use multi-year adult trapping data to help develop strategies that may be used by mosquito control districts to target *C. furens* populations where they are nuisance pests. Trap data from 2005 and 2007 identified seasonal peaks, high spatial heterogeneity, and substantial year-to-year variation, with an 88.3% reduction in trap captures between 2005 and 2007. These findings provide a foundation for integrated *Culicoides* management strategies where legal mandates, emerging pathogen risks, and taxpayer-driven nuisance complaints may justify expanded *Culicoides* control activities by Florida’s Mosquito Control Districts.

## Simple Summary

This study examined whether trapping data could identify persistent target areas for controlling the biting midge *Culicoides furens* on a residential island in Florida. Although trap catches varied strongly by season and location, no stable hot spots were found across years, limiting the value of fixed-site targeting. Abundance declined sharply from 2005 to 2007, and weather and seasonal timing were more useful than proximity to larval habitat for guiding control efforts. From the standpoint of area-wide management, these results highlight both the opportunities and limitations of managing *C. furens* under Florida’s legal and operational constraints and provide a baseline for future *Culicoides* control programs.

## 1. Introduction

*Culicoides* biting midges, known locally as “no-see-ums”, “sandflies” or “punkies”, are tiny bloodsucking flies (Diptera) in the family Ceratopogonidae that play a significant role in coastal Florida’s ecology, economy, and public health. In coastal areas such as Cedar Key, biting midges are seasonally extremely abundant, causing annoyance to residents and tourists. Their persistent biting activity interferes with beach recreation, fishing, hiking, biking and other outdoor activities. In addition to annoyance, their bites can cause severe itching and/or allergic reactions. The two species *Culicoides furens* (Poey) and *Culicoides mississippiensis* Hoffman are the main pest species at Cedar Key. Despite frequent requests from both residents and tourists, local mosquito control agencies cannot respond to these requests for relief either due to legal reasons or limited resources. Legally, all Florida mosquito control districts are governed by state rules (5E-13) and state laws (FL Ch-388) requiring an enabling act (CH 98-461) that is specific to the responsibilities of the district. Florida mosquito control districts are established to control arthropods of public health or nuisance importance, including mosquitoes and biting midges, under Chapter 388 of the Florida Statutes. However, district-specific enabling acts and limited revenues may restrict whether funds and staff time can realistically be allocated to biting midge control in addition to existing mosquito control operations.

Furthermore, mosquito control districts prioritize species that are primary vectors of human pathogens. Historically, biting midges have not been known to vector human pathogens in the United States. However, in 2024–2025 Oropouche virus (OROV) expanded its range into the Caribbean area, as shown with the virus isolated in blood plasma specimens collected from children in Haiti in 2014 [1]. Prior to 2025 OROV had been largely confined to the Amazon Basin in South America. OROV is a “sloth virus” that has been shown to be transmitted to humans primarily by the biting midge *Culicoides paraensis* (Goeldi). Florida has now documented 104 travel-related OROV cases from Cuba since the start of 2024 [2], highlighting the need for development of a best practices framework for a biting midge management program in the United States.

Establishing a program for biting midge management is challenging. Unlike mosquitoes, which typically breed in aquatic situations accessible to treatment, biting midges like *C. furens* breed in soil habitats associated with wetland ecosystems [3]. This restricts the ability to apply larval control measures effectively. With the absence of available biting midge larval treatments, vector control professionals must rely on adult biting midge control techniques that are modified from adult mosquito control protocols to protect the public from biting midges. Two separate studies [4,5] reported acceptable biting midge suppression for up to 6 weeks in a residential neighborhood with home treatments using the pyrethroid insecticide bifenthrin applied from a backpack sprayer as a low volume surface spray on foliage surrounding the homes. At a larger scale, ref. [6] reported that aerial applications with the organophosphate insecticide naled at maximum label rates provided an acceptable level of control for adult *C. furens* on Parris Island, South Carolina, when applied at peak midge flight activity, 2 h after sunset. Although aerial spraying with naled is very effective for mosquito control, without a complete integrated pest management (IPM) program to include midge larval control, adult biting midge control through aerial application was short lived, providing only 2–3 days of suppression of adult biting activity. These studies suggest that common mosquito control adulticide applications should be considered for suppression of biting midge populations, but that additional refinement and targeted interventions will be needed to successfully control biting midge populations.

Currently we are limited regarding area-wide biting midge control methods available for Florida’s mosquito control districts to protect their stakeholders against either nuisance biting or potential midge-borne disease. Furthermore, some of Florida’s mosquito control districts may not be statutorily allowed to control biting midges because their legal mandate is to control mosquitoes. In addition, all Florida mosquito control districts are governed by state rules (5E-13) and state laws (FL Ch-388) requiring an enabling act (CH 98-461) that is specific to the responsibilities of the district. Florida mosquito control districts are bound to these key statutes and may not have the flexibility to use revenues to control insects such as biting midges. Even if a district’s enabling act allowed for treatment of biting midges, they may not have the budget for additional insecticide and labor needed to control both mosquitoes and biting midges.

When developing control strategies for a residential area, treatments should be focused on the most productive *C. furens* breeding sites to ensure the most efficient and cost-effective measures are employed against adult *C. furens*. To provide proper suppression of *C. furens* populations, it will likely take multiple, innovative control strategies with appropriate timing in a well-managed IPM program to be successful. Thus, an understanding of the spatial and temporal distribution of biting midges along with an understanding of what environmental factors are correlated with midge abundance, such as tidal events, temperature, humidity, and rainfall, will be key in the development of a good management program. Such spatial and temporal data will provide vector control professionals with details of biting midge seasonality and distribution to help prescribe well-timed and precise treatments to achieve maximum population suppression.

The objective of this study is to describe the spatial and temporal patterns of *C. furens* on Rye Key Island, as well as the relationships between midge abundance and environmental factors, to provide insights to aid in the development of control strategies that may be used by mosquito control districts to target surveillance and treatments to biting midges. Using a combination of newly collected surveillance data and surveillance data previously collected by Lloyd [7], potential *C. furens* problem areas and population distribution trends within Rye Key, Florida, were evaluated in 2007. Analyses of these data were used in developing control strategies for *C. furens* based on IPM principles, including spatially and temporally targeted monitoring and interventions.

## 2. Materials and Methods

### 2.1. Study Area

Rye Key, Florida, is a 5.91 ha residential island surrounded by the Gulf of Mexico and extensive marsh inlets, located at the Northeast tip of Cedar Key, Florida. Rye Key was selected due to the large number of biting midges reported in [8], thus making it an ideal place for studying biting midge problems. In addition, surveillance data collected in the new study in 2007 are compared to data collected from Rye Key in a previous study conducted in 2005 [7].

The island and adjacent marshes support a variety of vertebrate hosts for *Culicoides*, including humans, dogs and cats around residences, and common coastal mammals, such as raccoons, that use the salt-marsh habitats. The broader Cedar Key area has a warm, humid subtropical climate, with mild winters (approximately 10–20 °C) and hot, wet summers (often 30–32 °C) and annual rainfall of roughly 1300–1500 mm with a pronounced summer–early autumn rainy season.

### 2.2. Biting Midge Trapping

*Culicoides furens* population surveillance began in April 2007 using sixteen Mosquito Magnet Liberty Plus ^®^ traps (WoodStream Corporation, Lititz, PA, USA) that were distributed in the same selected locations from a previous study performed in 2005 [7]. The sixteen traps were used to monitor adult midge populations. The Mosquito Magnet Liberty Plus is a propane powered trap designed to mimic a vertebrate host through the combustion of propane to generate heat, moisture, and CO_2_ to attract biting insects [9]. These traps can also be used with a variety of additional lures. In this study we used 1-octen-3-ol (octenol), a well-established kairomone for mosquitoes and biting flies [10,11]. Liberty Plus traps are cordless, have a push-button start, and have lights that indicate when the machine is operating or if service is needed. The trap manufacturer recommends the use of one trap per acre of land (~0.4 ha), placed in a suitable habitat near the mosquito larval source, to provide suppression of the mosquito population. For this study, trap sites were selected for an even distribution across Rye Key roughly following the manufacturers spacing recommendations, close to residences, easily accessible to maximize the potential benefits to the homeowner, and to provide efficient collection of trap captures.

*Culicoides furens* larvae are typically associated with *Spartina* and *Distichlis* marsh grass areas [3] and have previously been determined to be most abundant where these grasses are present [12]. To identify potential *C. furens* larval habitat and assist with biting midge trap placement, likely breeding habitats containing isolated groups of *Spartina* and *Distichlis* marsh grass were selected across the trapping area.

### 2.3. Experimental Protocol

The sixteen Liberty Plus traps placed on Rye Key were separated by ~0.40 ha each and left in their respective locations throughout the study (April through October 2007). Each trap was manually labeled, and GPS coordinates were recorded. Trap collections were conducted twice weekly (Tuesdays and Thursdays), during which nets containing captured insects were retrieved and replaced with clean nets. This schedule yielded a total of 832 individual trap collections in 2005 and 832 in 2007 across all 16 traps. Nets containing insects were placed into individual 3.8 L plastic Ziploc bags that were labeled with location, date, and trap identifier. Propane tanks used to power traps and produce CO_2_ were changed every 18 days. Attractant octenol cartridges used in traps were changed every 21 days.

Upon returning to the laboratory, all trap nets were placed into a walk-in cold room to immobilize the insects. Once the insects were immobile, they were placed into a 473 mL paper food carton, labeled, and stored in a −20 °C freezer until processed. After removal from the freezer, *Culicoides* were separated from the non-target insects using a 16-mesh copper screen, with a wire diameter of 0.28 cm. Most trap collections reached over a thousand specimens, with some trap collections exceeding 100,000 specimens, making the identification process so time consuming that timelines to process data for the study could not be met. To maintain trap collection processing times, a sub-sample of the original collection was occasionally taken. Specifically, when the total mass of *Culicoides* midges collected from a trap after sieving was less than 0.025 g (approximately <500 individuals), the entire sample was identified and counted. If the *Culicoides* captured weighed more than 0.025 g (>500 midges), a sub-sample of approximately 500 midges was taken from the total capture and weighed. The weight of the sub-sample was divided into the total captured weight, and the quotient was multiplied by the number of *Culicoides* identified and counted in the sub-sample. Biting midge samples were identified by species [3]. Although *C. mississippiensis* is an important nuisance species in the Cedar Key area generally, it was not detected in our Rye Key trap collections; all *Culicoides* identified from these samples were *C. furens*.

### 2.4. Weather Data

Weather data were gathered from 1 April 2005 to 31 October 2005 and from 1 April 2007 to 31 October 2007 to coincide with the dates of this study, and when *C. furens* populations are most abundant. The nearest National Oceanic and Atmospheric Administration (NOAA) station to Rye Key, National Data Buoy Center station CDRF-1 (29.136° N, 83.029° W), is approximately 3 km from the study site and provided monthly mean wind direction, wind speed, barometric pressure, and air temperature data.

### 2.5. Data Analyses

On the Western Coast of Florida, both *C. furens* and *C. mississippiensis* are recognized as important nuisance species. However, inspection and identification of trap collections during this study showed that *C. furens* comprised the vast majority of captured *Culicoides* at our Rye Key sites, with *C. mississippiensis* only collected sporadically during the two years of our study. Therefore, we restricted our quantitative analyses and management recommendations to *C. furens*.

Data analyses were conducted using Analyse-it Standard Edition for Microsoft Excel (version 2.0) (Analyse-it Software Ltd., Leeds, UK) and R studio (version 2.0) (R Core Team, Vienna, Austria). Visualization of midge abundance data over space and time are shown in vertical bar graphs. The total number of *C. furens* captured was calculated as described above per trap, and both monthly and annual numbers for 2005 were compared with 2007 to visualize *C. furens* abundance across Rye Key, FL, USA. We tested the normality of the distribution of *C. furens* captured per trap (L1-L16) in 2005 and in 2007, as well as the normality of the distribution of trap distance to the nearest larval habitat using Shapiro–Wilk tests. Neither parameter was normally distributed. Levene’s tests were conducted for both trap captures and distance between traps and larval habitats to assess homogeneity of variances. The test indicated unequal variances among traps in 2005 (F(15,816) = 4.91, *p* < 0.0001), traps in 2007 (F(15,816) = 3.19, *p* < 0.0001), distance between traps and larval habitat in 2005 (F(14,817) = 5.30, *p* < 0.0001), and distance between traps and larval habitat in 2007 (F(14,817) = 3.35, *p* < 0.0001). Thus, we chose to use non-parametric approaches for many of our analyses.

To determine if *C. furens* captured in 2005 tended to be more or less abundant than in 2007, a comparison of the annual average mean was calculated for *C. furens* captured in each of our 16 traps. To test whether traps with the most captures in 2005 were the same as the traps with the most captures in 2007 (i.e., hot spots vs. cold spots), all *C. furens* captured in each trap each year were combined then ranked from lowest to highest trap capture for 2005 and 2007. We compared these ranked lists between years compared using a Wilcoxon–Mann–Whitney test. Then we tested for consistency in hot spots and cold spots between 2005 and 2007 by testing for a relationship between the rank-sum value of a trap in 2005 versus the rank-sum value of the same trap in 2007 using Spearman’s rank correlation. A Steel–Dwass–Critchlow–Flingner pairwise comparison was used to identify trap locations that captured more or less *C. furens* within each year. We also analyzed monthly totals per trap and per year to determine when *C. furens* was most active and when activity declined each year. To determine if adult *C. furens* abundance in traps was correlated with larval habitat, Spearman’s rank correlation was used to measure the associations between annual average *C. furens* per trap night and distance between traps, as well as the correlation between traps and the distance to larval habitat to help establish guidelines for focused control strategies.

Spatial analyses were conducted in ArcGIS 9.2 (ESRI, Redlands, CA, USA). Feature classes were created for *C. furens* trap locations and for marsh grass (*Spartina* and *Distichlis*) measurements, and monthly mean trap captures for April–October 2005 and 2007 were mapped using the Geostatistical Analyst extension with graduated symbols to visualize spatial patterns in adult abundance [13]. Interpolation maps for each month and year were visually inspected to identify recurrent *C. furens* problem areas on Rye Key for the development of potential future management programs (Figure S1).

To test relationships between weather and midge abundance, which was collated by month in the original data sets, monthly weather data were compiled into a single dataset with the following monthly weather variables, mean wind direction, wind speed, barometric pressure, and air temperature, for a total of 14 monthly weather values, with 7 months sampled in 2005 and 2007 respectively. We then used a series of generalized linear models to test for relationships between midge abundance and the available weather variables using Akaike’s Information Criterion (AIC) to select the best model fit for weather–abundance relationship of *C. furens* on Rye Key (Table S1).

## 3. Results

**Figure 1:**
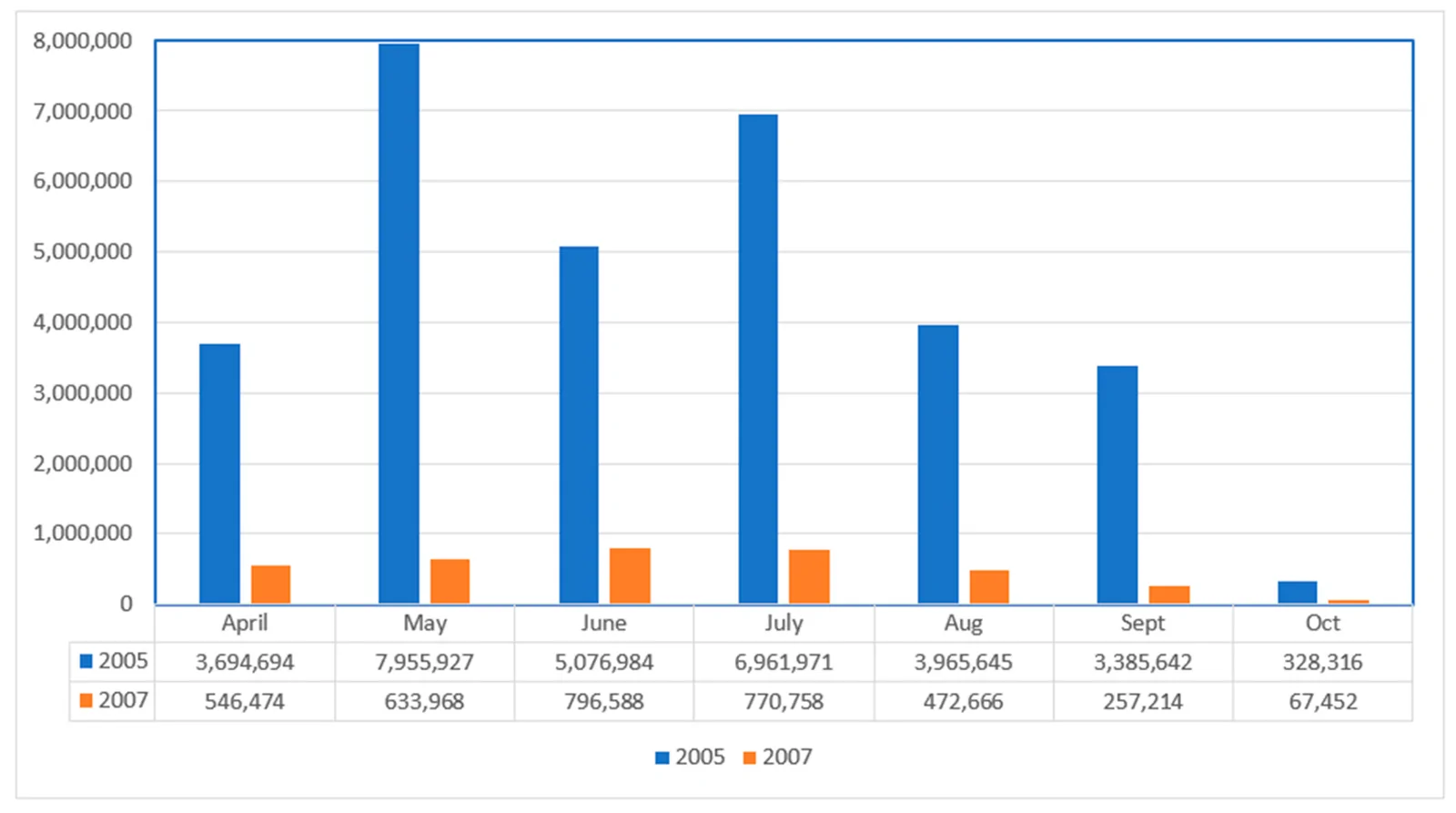
Monthly totals of *Culicoides furens* captured by all 16 traps on Rye Key, Florida, in 2005 and 2007. Bars show the total number of midges collected each month (April–October) in 2005 (blue) and 2007 (orange), illustrating consistently higher captures and earlier, stronger seasonal peaks in 2005. Each monthly bar shows the total number of *C. furens* captured across 8 trap collections, with 2 collections each week.

In April–October 2005 and April–October 2007, all 16 traps on Rye Key, Florida, captured 32,258,666 and 3,760,283 *C. furens*, respectively. When comparing the average numbers captured per trap, totals in 2005 were 88.3% greater than in 2007 (Wilcoxon–Mann–Whitney test, *p* < 0.0001). Monthly trap captures were consistently higher in 2005, with peaks typically occurring from May to July across most locations, indicating strong early to mid-summer abundance (Figure 1). In 2007, captures were much lower in all months, and seasonal peaks were not as pronounced as they were in 2005. In 2005, multiple traps (L1, L10, L12, and L16) captured high mid-summer totals in the hundreds of thousands to over a million, while late-season (October) counts declined sharply, indicating the end of the season for *C. furens* (Table 1). In 2007, monthly captures rarely exceeded tens of thousands throughout the season, but October captures remained low following the end of season trends reported in 2005. Comparing traps across years, total seasonal reductions exceeded 80–90% at most locations, although a few traps and months showed localized increases in abundance at those sites (L2–L8) (Table 1).

**Figure 2:**
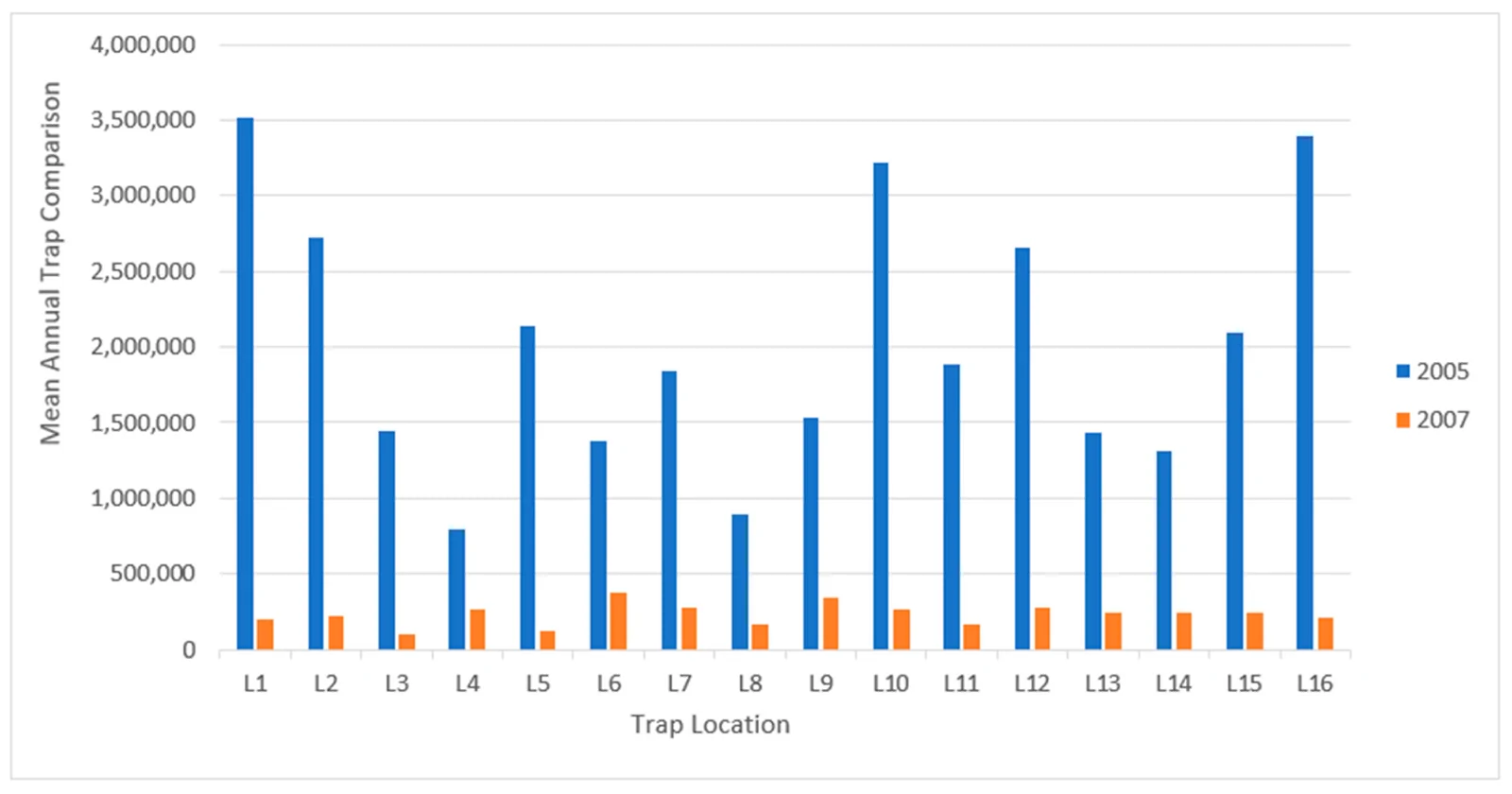
Monthly total *Culicoides furens* captured at 16 trap locations (L1–L16) on Rye Key, Florida, in 2005 and 2007. Bars show the monthly total number of midges captured at each location in 2005 (blue) and 2007 (orange), illustrating spatial variation among traps and a consistent reduction in abundance across all locations in 2007.

**Figure 3:**
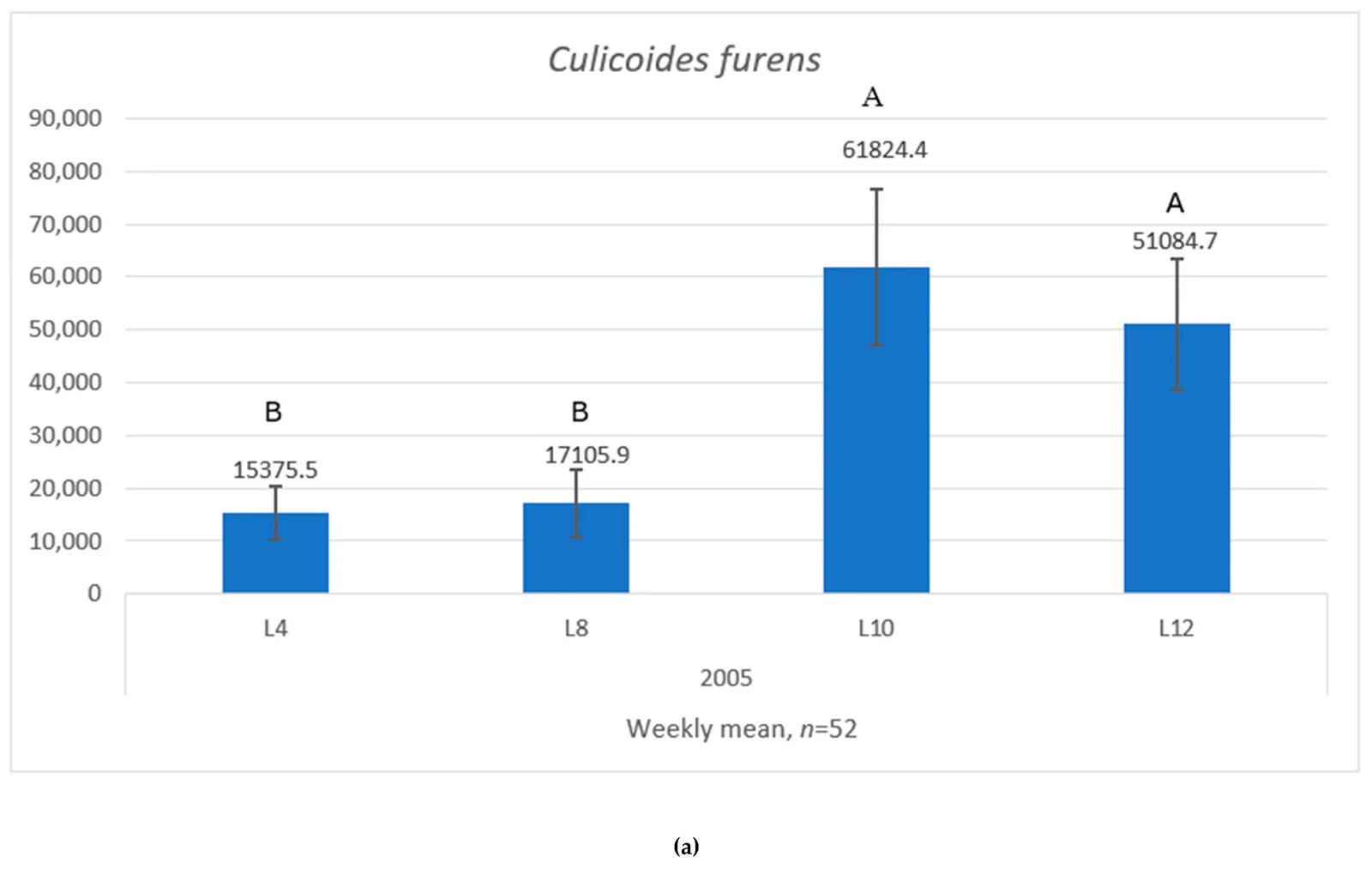

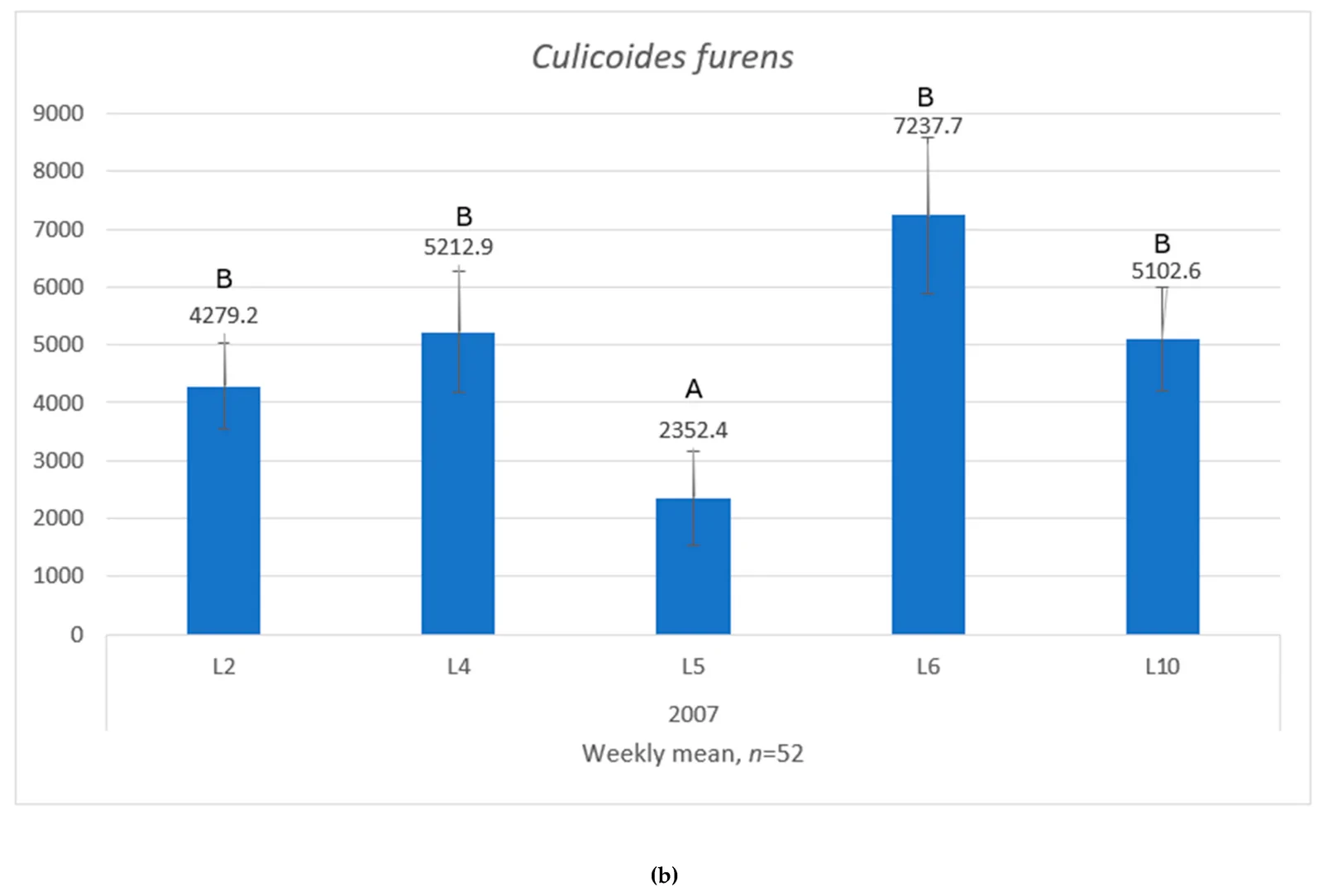
(**a**) Mean weekly *Culicoides furens* captured per trap at locations L4, L8, L10, and L12 on Rye Key, Florida, during 2005 (*n* = 52 trap-weeks per location). Bars show means ± SE; different letters indicate significantly different trap means within the year based on Steel–Dwass–Critchlow–Fligner pairwise comparisons. (**b**) Mean weekly *Culicoides furens* captured per trap at locations L2, L4, L5, L6, and L10 on Rye Key, Florida, during 2007 (*n* = 52 trap-weeks per location). Bars show means ± SE; different letters indicate significantly different trap means within the year based on Steel–Dwass–Critchlow–Fligner pairwise comparisons.

To identify areas within Rye Key to concentrate *C. furens* control efforts, we tested the extent to which there were “hot spots” and “cold spots” among trap captures in 2005 and 2007 for all 16 traps (Figure 2). There was clear evidence of some traps capturing significantly more *C. furens* than other traps in both 2005 (Kruskal–Wallis, df 15, *p* = 0.0004) and 2007 (Kruskal–Wallis, df 15, *p* = 0.0006) (Figure 3a,b, Table 1). Specifically, in 2005, trap 8 (L8) had significantly fewer *C. furens* captured than traps L10 (Steel–Dwass–Critchlow–Flingner pairwise comparisons, *p* = 0.0236) and L12 (*p* = 0.0083) (Figure 3a). Traps L10 and L12 also had a greater number of *C. furens* captured than trap L4 (*p* = 0.0261). In 2007, trap 5 had significantly fewer *C. furens* captured than traps L6 (*p* = 0.0052), L10 (*p* = 0.0415), and L2 (*p* = 0.0327) (Figure 3b). Although there is evidence of significant differences among traps within each year, the differences in trap capture were not the same for 2005 and 2007 (Spearman’s rank correlation, r = −0.035, *p* = 0.90), limiting the ability to prescribe targeted *C. furens* control measures because there were not clear and repeatable “hot spots” or “cold spots” between traps from 2005 and 2007 (Figure 3a,b).

To determine whether there was a relationship between the distance a trap covered relative to the larval habitat and trap capture, we tested for a relationship between the straight-line distance from each trap location to the closest larval habitat in both 2005 and 2007. There was no relationship between larval habitat distance to a trap and the number of *C. furens* captured in that trap in 2005 (Spearman’s correlation, r = −0.144, *p* = 0.59) or in 2007 (r = −0.225, *p* = 0.40).

When testing for relationships between weather variables and monthly *C. furens* midge abundance, the best-supported generalized linear model (AIC = 35.25) showed significant positive associations between midge abundance and pressure, temperature, and wind direction; a weaker positive association with wind speed; and a strong negative effect of year (glm(formula = Abundance_mil ~ Pressure + Temp + Wind_Dir + Wind_Speed + Year + 1, family = Gamma(link = “log”)). In this model, midge abundance increased significantly as the mean wind direction shifted toward southerly sectors and with higher wind speeds, higher barometric pressures, and higher temperatures, after accounting for year effects (Wald chi-square, df 8, *p* = 0.046). However, a simpler model (glm(formula = Abundance_mil ~ Wind_Dir, family = Gamma(link = “log”)) containing only wind direction also showed a significant positive association with *C. furens* abundance with a similar model fit (AIC = 37.26) (Wald chi-square, df 13, *p* = 0.014).

## 4. Discussion

This study demonstrated a large reduction in *C. furens* captured between 2005 and 2007, with 2005 trap collections exceeding those in 2007 by 88.3% across the 16 traps. This decline was consistent across most traps and months, with seasonal reductions typically exceeding 80–90% in total trap captures, indicating an observed area-wide population decline across Rye Key rather than a localized effect at specific trap sites. There was high variability in *C. furens* captures among trap locations within each year, with no repeatability of hot spots between 2005 and 2007, which limits targeted control efforts and suggests an area-wide approach will be needed. Extending adult surveillance across additional years could help determine whether any locations consistently function as hot or cold spots and improve the ability to design spatially targeted control strategies over the long term. From an operational perspective, the lack of temporal stability in hot spots implies that control strategies based on fixed trap locations alone may provide poor insights into control efforts over multiple seasons and that additional surveillance, such as larval counts, may be needed to target control efforts and make an impact on *C. furens* biting pressure. In 2005, trap captures showed early to mid-summer peaks (May to August) at most trap locations, often reaching hundreds of thousands to more than one million *C. furens* per month, followed by an October decline that marked a declining season. In 2007, similar seasonal peaks and declines were observed, with monthly totals rarely exceeding tens of thousands, suggesting that the seasonal abundance of *C. furens* was stable even with an overall reduction in abundance.

Given that *C. furens* larvae are typically thought to be associated with *Spartina* and *Distichlis* marsh grasses [3,12], we expected to find a relationship between distance from larval habitat and trap abundance on Rye Key. However, we detected no relationship between distance to larval habitat and trap captures. This lack of association suggests that simple linear measurements for distance from trap locations to larval habitat were insufficient for identifying locations to concentrate control efforts on Rye Key and that an area-wide approach to control will be needed in this location.

Mean monthly air temperatures and wind speeds during the April–October sampling periods in 2005 and 2007 were broadly similar, with only modest differences between years, and thus weather parameters alone are unlikely to explain the much lower abundance of *C. furens* in 2007 compared to 2005. In both years midge abundance was greater when there was high barometric pressure and warmer temperatures, which is to be expected. Perhaps more interesting is that in both years wind direction explained much of the variance in midge abundance. Specifically, the highest *C. furens* abundances were observed in months that had predominantly southerly to south–southwesterly winds (approximately 150–195°) in 2005 and 2007. Thus, the directionality of the prevailing winds may be considered when timing surveillance and control interventions on Rye Key.

### 4.1. Trap-Mediated Population Suppression

The intended design for this study utilized traps as surveillance to identify problem areas and help direct control efforts for *C. furens* population suppression. However, one possibility is that our heavy trapping in 2005 led to the population decline that was observed 2 years later in 2007 because other *Culicoides* trapping studies have also shown substantial but temporary population declines [8,14]. Yet, these reductions are typically not permanent once the traps have been removed [15]. Our traps continuously removed large numbers of host-seeking *C. furens* over the season (April–October), with several traps capturing millions of individuals in 2005, and the reduction in trap capture reported in 2007 occurred without changes in trapping locations, trap design, or sampling protocol. Although the percent reduction cannot be directly attributed to the trap capture, the pattern aligns with the broader literature on mass trapping of adult *Culicoides* and the Ecological trap theory where altered cues (insect traps) lead insects to choose low-quality habitats that contribute to population decline [16].

### 4.2. Operational Implications

The seasonal dynamics observed in this study suggest that control strategies for *C. furens* should begin in April when populations are on the rise, maximizing efforts during the May to August peak, with diminished efforts needed as captures decline toward the end of the season in October. On residential islands, this treatment schedule would provide the highest likelihood of operational impact. Because no soil insecticides are currently available for use in these substrates and regulatory constraints prevent physical alteration of the salt marsh, larval *C. furens* habitats cannot be directly treated. The lack of control options for *C. furens* larval habitat force control efforts to rely on adult *C. furens* control to manage the population. Adult *C. furens* surveillance must be conducted to direct control strategies, and in an island environment, this study brings to light the difficulty in identifying repeatable hot spots for small-scale precise adult *C. furens* treatments, like those that might be contracted by individual homeowners. The results observed by surveying larval habitats close to adult trap locations make it clear that identifying problem areas to target adult *C. furens* treatments with larval habitat alone does not work, likely due to the immense amount of larval habitat that surrounds the island. Instead, operational plans could emphasize a reduced number of adult surveillance traps placed with deployment efficiency in mind and permitted access to meet the required treatment thresholds needed for an adulticide treatment by air or ground. Alternatively, the reduction in *C. furens* captured between 2005 and 2007 across 16 traps, deployed at the manufacturer’s recommended density, suggest that intensive season-long trapping can coincide with substantial area-wide declines in adult abundance on an isolated island, even without larviciding or adulticiding. Furthermore, traps can serve dual roles as surveillance and control, continuously removing adult *C. furens* throughout the season. On small islands where residences are concentrated near shorelines, prioritizing trap placement close to homes and docks can maximize stakeholders’ benefit from some control while also generating surveillance data.

Adulticide applications can be used to temporarily manage adult *C. furens* populations on small residential islands but must be timed to coincide with peak activity around dusk to maximize spray efficiency. Organophosphate products, such as naled, have produced short-term knockdown of *C. furens* [17], but these treatments are expensive and should be limited due to budget and label restrictions. Pyrethroid low-volume barrier applications can further extend protection around residences [5], but adulticides are best implemented within an integrated pest management framework that includes sustained adult surveillance and, where feasible, additional control techniques, such as trapping, because adulticide applications are generally effective less than a week before populations rebound [17,18,19].

Looking ahead, continued monitoring of *C. furens* on Rye Key should combine adult surveillance traps with targeted larval assessments in *Spartina*/*Distichlis* marshes with wind direction and speed data so we can more fully understand the effects of these environmental parameters on *C. furens* abundance. Building on the 2005 and 2007 data, such integrated monitoring would help elucidate when and where residents experience peak biting pressure and provide a framework for an island-scale integrated pest management program.

## 5. Conclusions

From the standpoint of an area-wide control program, this study on Rye Key Island highlights both opportunities and limitations for managing *C. furens* under legal and operational constraints in Florida. The threat of invasive human pathogens to Florida, such as Oropouche virus, elevates *Culicoides* from a nuisance concern to a potential public health threat, underscoring the need to proactively develop management programs. In addition, many coastal communities are disrupted by severe biting nuisance caused by *Culicoides* reducing outdoor recreation, property use, and tourism that require control. These high levels of biting pressure generate substantial numbers of citizen complaints that increase political pressure for relief. In this study, multi-year trapping abundance data did not identify target areas for control, but they are valuable for characterizing seasonal dynamics and supporting island-scale, area-wide *Culicoides* management decisions. Results from this study provide a baseline that will assist entities preparing a *C. furens* management program that can also be expanded to include other *Culicoides* species.

## Figures and Tables

**Table 1:** Monthly adult *Culicoides furens* counts and percent reduction at the trap locations L1–L16 for 2005 (baseline) and 2007. Each monthly total shows the total number of *C. furens* captured across 8 trap collections, with 2 collections each week.

Trap	Year	Apr	May	Jun	Jul	Aug	Sept	Oct	Total
L1	2005	186.6K	1.14M	877.3K	646.9K	394.9K	160.6K	112.6K	3.52M
	2007	23.8K	29.0K	47.6K	82.6K	13.3K	7.8K	0.5K	204.6K
	% reduction	87.3	97.5	94.6	87.2	96.6	95.2	99.5	94.2
L2	2005	575.3K	832.9K	76.6K	741.7K	41.1K	451.4K	5.0K	2.72M
	2007	49.8K	36.4K	52.6K	3.8K	55.5K	17.4K	7.0K	222.5K
	% reduction	91.3	95.6	31.3	99.5	−35	96.2	−39.2	91.8
L3	2005	127.2K	189.1K	152.8K	191.2K	544.2K	226.6K	10.7K	1.44M
	2007	14.0K	33.0K	6.2K	11.7K	13.0K	15.4K	11.1K	104.4K
	% reduction	89	82.5	95.9	93.9	97.6	93.2	−4	92.8
L4	2005	256.9K	282.2K	18.5K	163.0K	57.6K	18.4K	2.2K	798.8K
	2007	29.2K	57.2K	11.9K	117.2K	29.8K	21.1K	4.7K	271.1K
	% reduction	88.7	79.7	35.5	28.1	48.2	−14.8	−112.4	66.1
L5	2005	177.7K	216.0K	917.3K	291.4K	509.3K	18.5K	4.1K	2.13M
	2007	18.2K	45.9K	4.0K	2.7K	4.5K	38.1K	8.9K	122.3K
	% reduction	89.7	78.7	99.6	99.1	99.1	−105.6	−118.7	94.3
L6	2005	202.5K	394.9K	265.1K	389.1K	82.5K	31.1K	9.4K	1.37M
	2007	32.0K	122.9K	118.0K	42.0K	27.9K	33.1K	0.5K	376.4K
	% reduction	84.2	68.9	55.5	89.2	66.1	−6.5	95.1	72.6
L7	2005	115.5K	536.9K	336.1K	456.6K	59.3K	329.3K	11.1K	1.84M
	2007	35.9K	21.3K	81.4K	21.6K	106.3K	9.0K	1.7K	277.2K
	% reduction	68.9	96	75.8	95.3	−79.3	97.3	84.9	85
L8	2005	154.2K	181.7K	26.6K	381.1K	111.3K	22.5K	12.2K	889.5K
	2007	16.6K	16.8K	62.0K	28.9K	19.1K	25.6K	2.1K	171.1K
	% reduction	89.3	90.8	−133.2	92.4	82.8	−14.1	82.9	80.8
L9	2005	168.6K	454.7K	222.1K	168.1K	148.6K	359.4K	14.9K	1.54M
	2007	44.0K	73.6K	89.0K	85.0K	23.7K	25.7K	3.2K	344.2K
	% reduction	73.9	83.8	59.9	49.5	84	92.9	78.4	77.6
L10	2005	486.3K	1.09M	571.7K	375.3K	162.9K	512.9K	16.6K	3.21M
	2007	59.3K	32.5K	62.6K	35.4K	51.9K	20.1K	3.4K	265.3K
	% reduction	87.8	97	89	90.6	68.1	96.1	79.8	91.8
L11	2005	105.4K	429.2K	631.5K	374.9K	172.9K	153.7K	17.6K	1.89M
	2007	17.6K	15.6K	50.0K	39.9K	14.9K	15.6K	13.4K	167.0K
	% reduction	83.3	96.4	92.1	89.4	91.4	89.8	24	91.1
L12	2005	126.0K	582.1K	313.4K	507.7K	614.2K	438.9K	73.4K	2.66M
	2007	15.9K	41.4K	97.2K	77.1K	32.9K	9.7K	4.0K	278.2K
	% reduction	87.4	92.9	69	84.8	94.7	97.8	94.5	89.5
L13	2005	504.2K	440.3K	59.1K	245.7K	103.3K	64.7K	15.3K	1.43M
	2007	51.1K	40.0K	18.3K	105.0K	21.9K	9.8K	2.6K	248.7K
	% reduction	89.9	90.9	69	57.3	78.8	84.9	82.9	82.6
L14	2005	448.2K	136.9K	295.6K	371.1K	30.8K	27.0K	5.1K	1.31M
	2007	51.1K	40.0K	18.3K	105.0K	21.9K	9.8K	2.6K	248.7K
	% reduction	88.6	70.8	93.8	71.7	28.9	63.7	48.5	81.1
L15	2005	63.9K	564.7K	95.9K	786.4K	37.8K	539.4K	8.0K	2.10M
	2007	54.9K	25.1K	43.9K	105.3K	4.8K	7.5K	3.0K	244.7K
	% reduction	14	95.6	54.2	86.6	87.2	98.6	62	88.3
L16	2005	150.4K	667.7K	244.1K	1.25M	1.01M	53.7K	22.5K	3.40M
	2007	28.0K	28.3K	69.2K	32.3K	39.8K	15.6K	0.8K	214.0K
	% reduction	81.4	95.8	71.6	97.4	96.1	70.9	96.5	93.7

Negative values indicate higher counts in 2007 than in 2005. Numbers are expressed in thousands (K) and millions (M) notation.

## Data Availability

The raw data supporting the conclusions of this article will be made available by the authors on request.

## Supplementary Materials

The following supporting information can be downloaded at: https://www.mdpi.com/article/10.3390/insects17060653/s1, Figure S1. Graduated Symbol interpolation map for monthly mean *Culicoides furens* abundance captured in June 2005. This map contains eight classes, with 2000 minimum to 105,000 maximum numbers captured. Areas outlined in red are salt-marsh grass generally associated with *C. furens* larval habitat. Map generated using ARC GIS 9.2. Table S1. Model selection results for *Culicoides furens* abundance based on candidate generalized linear models including atmospheric pressure, temperature, wind direction, wind speed, and year as predictors.

## References

[1] Elbadry M.A., Durães-Carvalho R., Blohm G.M., White S.K., van de Guchte A., Stephenson C.J., Loeb J.C., Telisma T., Chavannes S., De Rochars V.M.B. (2021). Orthobunyaviruses in the Caribbean: Melao and Oropouche virus infections in school children in Haiti in 2014. PLoS Neglected Trop. Dis..

[2] Florida Department of Health (FDOH) (2024). Florida Arbovirus Surveillance, Week 52: International Travel-Associated Oropouche Fever Cases, Florida, 2024.

[3] Blanton F.S., Wirth W.W. (1979). The Sand Flies (Culicoides) of Florida (Diptera: Ceratopogonidae). Arthropods Of Florida and Neighboring Land Areas.

[4] Lloyd A.M., Kline D.L., Bernier U. (2021). Field evaluation of Talstar (bifenthrin) residential barrier treatments alone and in conjunction with Mosquito Magnet Liberty Plus traps in Cedar Key, Florida. J. Fla. Mosq. Control Assoc..

[5] Standfast H.A., Fanning I., Maloney L., Purdie D., Brown M. (2003). Field evaluation of Bistar environmental health insecticide as an effective insecticide harborage treatment for biting midges (*Culicoides*) and mosquitoes infesting peridomestic situations in an urban environment. Bull. Mosq. Control Assoc..

[6] Breidenbaugh M.S., de Szalay F.A. (2010). Effects of aerial applications of naled on nontarget insects at Parris Island, South Carolina. Environ. Entomol..

[7] Lloyd A.M. (2006). Evaluation of Mosquito Magnet Traps for Biting Midge Control on Rye Key, Florida. Master’s Thesis.

[8] Lloyd A.M., Kline D.L., Hogsette J.A., Kaufman P.E., Allan S.A. (2008). Evaluation of two commercial traps for the collection of *Culicoides* (Diptera: Ceratopogonidae) in Florida, USA. J. Am. Mosq. Control Assoc..

[9] Kline D.L. (2002). Evaluation of various models of propane-powered mosquito traps. J. Vector Ecol..

[10] Takken W., Kline D.L. (1989). Carbon dioxide and 1-octen-3-ol as mosquito attractants. J. Am. Mosq. Control Assoc..

[11] Kemme J.A., van Essen P.H., Ritchie S.A., Kay B.H. (1993). Response of mosquitoes to carbon dioxide and 1-octen-3-ol in southeast Queensland, Australia. J. Am. Mosq. Control Assoc..

[12] Wood J.R., Kline D.L. (1989). Seasonal and spatial distribution of *Culicoides furens* and *C. mississippiensis* (Diptera: Ceratopogonidae) in a Florida mangrove swamp. Environ. Entomol..

[13] Longley P.A., Goodchild M.F., Maguire D.J., Rhind D.W. (2001). Geographic Information Systems and Science.

[14] Cilek J.E., Kline D.L., Hallmon C.F. (2003). Evaluation of a novel removal trap system to reduce biting midge (Diptera: Ceratopogonidae) populations in Florida backyards. J. Vector Ecol..

[15] Carpenter S., Mellor P.S., Torr S.J. (2008). Control techniques for *Culicoides* biting midges and their application in the U.K. and northwestern Palaearctic. Med. Vet. Entomol..

[16] Robertson B.A., Rehage J.S., Sih A. (2013). Ecological novelty and the emergence of evolutionary traps. Trends Ecol. Evol..

[17] Breidenbaugh M.S., Foley E.H., Brooks C., Reeves W.K. (2020). Nighttime aerial sprays for control of crepuscular biting midges in South Carolina. J. Am. Mosq. Control Assoc..

[18] Linley J.R., Reiter P., Petersen J.L. (1987). Evaluation of naled applied as a thermal fog against *Culicoides furens* and other pestiferous midges in Florida. J. Am. Mosq. Control Assoc..

[19] Breidenbaugh M.S., Haagsma K.A., Linthicum K.J. (2009). Seasonal and diel patterns of biting midges (Diptera: Ceratopogonidae) and implications for their control at a coastal bird rookery in Florida. J. Am. Mosq. Control Assoc..

