# Correction: Lloyd et al. Can Trapping Abundance Data Be Used to Identify Persistent Target Areas for *Culicoides* Biting Midge Control Efforts? *Insects* 2026, *17*, 653

**DOI:** 10.3390/insects17101042

**Published:** 2026-10-10

**Authors:** Aaron M. Lloyd, Daniel L. Kline, Karen E. McKenzie, Daniel A. Hahn

**Affiliations:** 1 Lee County Mosquito Control District, Lehigh Acres, FL 33971, USA; 2 United States Department of Agriculture-Agricultural Research Service- Center for Medical, Agricultural and Veterinary Entomology, Gainesville, FL 32608, USA; dan.kline@usda.gov; 3 Contract Entomologist, Melbourne, FL 32904, USA; drkemckenzie@gmail.com; 4 Entomology and Nematology Department, University of Florida, Gainesville, FL 32611, USA; dahahn@ufl.edu

## Text Correction

There was an error in the original publication [1]. A sentence was changed in the Introduction Section, page 2, second paragraph. In the Introduction on page 2, second paragraph of the manuscript, the following sentence:

“Historically, biting midges have not been known to vector human or animal pathogens.”, should be changed to the following: “Historically, biting midges have not been known to vector human pathogens in the United States.” 

The authors state that the scientific conclusions are unaffected. This correction was approved by the Academic Editor. The original publication has also been updated.
