# Supplementary material for: Can Trapping Abundance Data Be Used to Identify Persistent Target Areas for Culicoides Biting Midge Control Efforts?
Source: Insects. 2026 Jun 20;17(6):653. doi: 10.3390/insects17060653 (PMC13300559; doi:10.3390/insects17060653)
Supplement: Supplementary file 1 [file insects-17-00653-s001.zip › insects-4350956-supplementary.pdf]

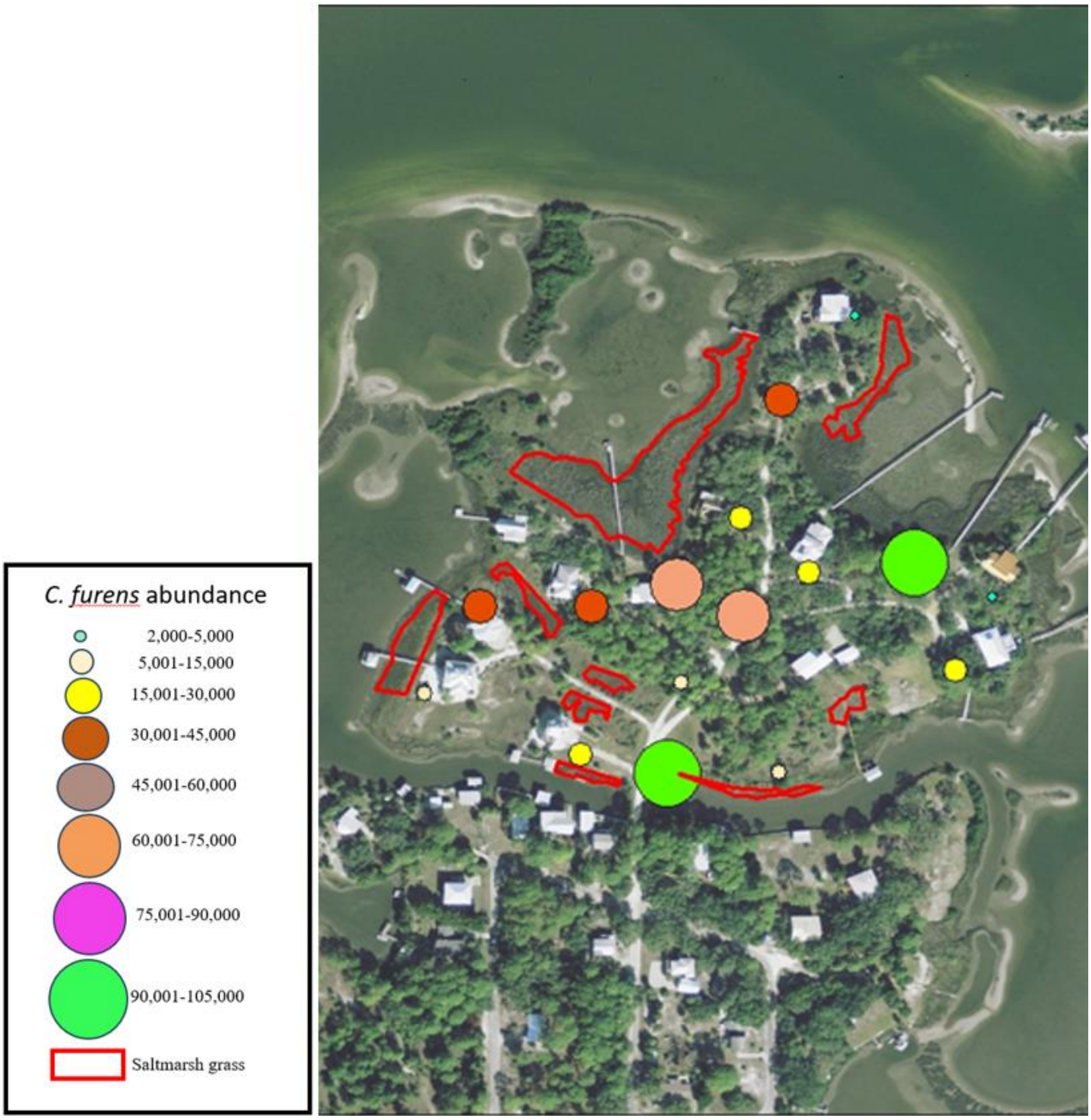

Figure S1. *Graduated Symbol interpolation map for monthly mean Culicoides furens abundance captured in June 2005. This map contains eight classes with 2,000 minimum to 105,000 maximum number captured. Areas outlined in red are saltmarsh grass generally associated with C. furens larval habitat. Map generated using ARC GIS 9.2.*

Table S1. Model selection results for *Culicoides furens* abundance based on candidate generalized linear models including atmospheric pressure, temperature, wind direction, wind speed, and year as predictors.

Model	Intercept	Pressure	Temp	Wind dir	Wind speed	Year	df	logLik	AIC	ΔAIC
**1**	2037.12	0.08	0.15	0.03	1.01	-1.06	7	-10.62	35.25	0.0
**2**	2279.56	NA	NA	0.02	NA	-1.14	4	-14.63	37.26	2.01
**3**	2011.29	0.06	0.07	0.02	NA	-1.03	6	-12.69	37.38	2.13
**4**	2098.94	0.04	NA	0.02	NA	-1.07	5	-13.87	37.73	2.48
**5**	2248.32	NA	0.06	0.02	NA	-1.12	5	-13.99	37.97	2.72
**6**	2321.12	NA	0.09	0.02	0.5	-1.16	6	-13.55	39.1	3.85
**7**	2285.9	NA	NA	0.02	0.05	-1.14	5	-14.62	39.25	4.0
**8**	2106.91	0.05	NA	0.02	0.15	-1.08	6	-13.81	39.62	4.37
**9**	2242.66	NA	NA	NA	NA	-1.12	3	-18.34	42.68	7.43
**10**	2225.9	NA	NA	NA	-0.57	-1.11	4	-17.69	43.37	8.12
**11**	2078.35	0.04	NA	NA	NA	-1.06	4	-17.98	43.96	8.71
**12**	2239.67	NA	0.04	NA	NA	-1.12	4	-18.14	44.29	9.04
**13**	2106.97	0.03	NA	NA	-0.5	-1.07	5	-17.46	44.91	9.66
**14**	2040.94	0.05	0.05	NA	NA	-1.04	5	-17.63	45.26	10.01
**15**	2225.7	NA	0.0	NA	-0.56	-1.11	5	-17.69	45.37	10.12
**16**	2086.91	0.04	0.02	NA	-0.41	-1.06	6	-17.42	46.85	11.6
**17**	-85.36	0.08	NA	0.03	NA	NA	4	-24.9	57.8	22.55
**18**	-3.77	NA	NA	0.03	NA	NA	3	-25.99	57.97	22.72
**19**	-100.35	0.09	0.12	0.03	NA	NA	5	-24.04	58.09	22.84
**20**	0.96	NA	NA	NA	NA	NA	2	-27.13	58.25	23.0
**21**	-84.05	0.08	NA	NA	NA	NA	3	-26.21	58.42	23.17
**22**	-6.72	NA	0.1	0.03	NA	NA	4	-25.54	59.08	23.83
**23**	4.09	NA	NA	NA	-0.75	NA	3	-26.78	59.56	24.31
**24**	-1.08	NA	NA	0.02	-0.51	NA	4	-25.83	59.67	24.42
**25**	-82.18	0.08	NA	0.03	-0.15	NA	5	-24.89	59.77	24.52
**26**	-114.67	0.1	0.14	0.03	0.42	NA	6	-23.93	59.86	24.61
**27**	-94.87	0.09	0.06	NA	NA	NA	4	-25.94	59.87	24.62
**28**	-0.0	NA	0.04	NA	NA	NA	3	-27.04	60.08	24.83
**29**	-73.96	0.08	NA	NA	-0.4	NA	4	-26.11	60.21	24.96
**30**	-5.01	NA	0.09	0.03	-0.29	NA	5	-25.49	60.97	25.72
**31**	3.63	NA	0.01	NA	-0.7	NA	4	-26.77	61.54	26.29
**32**	-93.97	0.09	0.06	NA	-0.02	NA	5	-25.94	61.87	26.62

Models are ranked by Akaike's information criterion (AIC), with ΔAIC indicating the difference from the best supported model and NA denoting parameters not included in each model. Abbreviations: Temp, temperature; Wind dir, wind direction; Wind speed, wind speed; df, degrees of freedom; logLik, model log likelihood; AIC, Akaike information criterion; ΔAIC, difference from the best model.
